# Patient’s point of view on the use of telemedicine in multiple sclerosis: a web-based survey

**DOI:** 10.1007/s10072-021-05398-6

**Published:** 2021-07-20

**Authors:** Doriana Landi, Marta Ponzano, Carolina Gabri Nicoletti, Gaia Cola, Gianluca Cecchi, Alfonso Grimaldi, Giorgia Mataluni, Nicola Biagio Mercuri, Maria Pia Sormani, Guglielmo Pacileo, Girolama Alessandra Marfia

**Affiliations:** 1 Multiple Sclerosis Research and Clinical Unit, Tor Vergata University Hospital, Viale Oxford 81, 00133 Rome, Italy; 2 Department of Systems Medicine, Tor Vergata University, Rome, Italy; 3 Department of Health Sciences, University of Genoa, Genoa, Italy; 4 Centre of Research On Health and Social Care Management, Unit of Neurology, SDA Bocconi School of Management, Bocconi University, Milan, Italy; 5 IRCCS Istituto Neurologico Mediterraneo (NEUROMED), Pozzilli, IS Italy

**Keywords:** Telemedicine, Televisit, Multiple sclerosis, Remote monitoring, Survey, COVID-19 pandemic

## Abstract

Restrictions in the access to healthcare facilities during COVID-19 pandemic have raised the need for remote monitoring of chronic medical conditions, including multiple sclerosis (MS). In order to enable the continuity of care in these circumstances, many telemedicine applications are currently tested. While physicians’ preferences are commonly investigated, data regarding the patients’ point of view are still lacking. We built a 37 items web-based survey exploring patients’ propensity, awareness, and opinions on telemedicine with the aim to evaluate the sustainability of this approach in MS. Analysing 613 questionnaires out of 1093 that were sent to persons with MS followed at the Multiple Sclerosis Center of Tor Vergata University, Rome, we found that more than half of respondents (54%) were open to having a televisit. Propensity toward telemedicine significantly depended on having a higher income (*p* = 0.037), living farther from the center (*p* = 0.038), using computer and tablet (*p* = 0.010) and using the Internet for other remote activities (*p* < 0.001), conversely it was not influenced by any specific disease characteristics (i.e. degree of disability). The main advantages and disadvantages of televisit reported by participants were respectively saving time (70%) and impossibility to measure physical parameters (71%). Although the majority of respondents are in favour of televisit, so far this approach is restricted to those displaying better socioeconomic conditions and higher familiarity with technology. Implications of the study are that telemedicine platforms should be better tailored to patients’ demands in order to spread the use of telemedicine, to enhance usability and to increase patients’ adherence.

**Supplementary Information:**

The online version contains supplementary material available at 10.1007/s10072-021-05398-6.

## Introduction

COVID-19 pandemic, which was declared on March 11th, 2020, has had a profound impact on the organization of the entire healthcare system. At the peak of the pandemic, most of the healthcare resources were used for the management of patients with COVID-19, and the access to the hospital was restricted to medical emergencies, according to Governments’ recommendations. Nevertheless, people were also afraid of getting infected with Sars-Cov2 at the hospital or through contacts with healthcare professionals. Therefore, for those patients with chronic diseases, such as multiple sclerosis (MS), COVID-19 pandemic has had not only direct, but also indirect impact, affecting the continuity and quality of their routine care [1]. In Italy, such as in many other countries, in fact, the majority of the face-to-face encounters were cancelled; patients and doctors had to quickly adapt to such mutated conditions and find new ways to ensure the continuity of care.

When possible, in-person visits were replaced by telephone visit or video visit, with different levels of doctors and patients’ satisfaction, mostly depending on the type of visit (audio/video), patient’s age, diagnosis, socioeconomic status, quality of the available technological devices, and presence of a caregiver [2–10].

Due to the pandemic, televisit was proposed by clinicians to all patients, without any predetermined selection based on sociodemographic or medical or other meaningful variables. Nevertheless, in the case of patients with MS, for example, specific clinical features, such as the degree of neurological disability or the type of disease modifying therapy, may impact on the attitude of MS patients toward telemedicine.

In this study we aimed to explore, through a web-based survey, the propensity of MS patients to telemedicine and the demographic, socioeconomic, clinical, and logistic variables associated with higher/lower propensity to telemedicine.

## Methods

### Setting

The MS centre of Tor Vergata University hospital assists a large number of patients (> 1500) with MS and other demyelinating disorders living in a vast area of Rome and its south east suburbs, with a minority of patients living in other Italian regions and abroad. In normal circumstances at least two in-person visits/year are scheduled for routine disease monitoring for each patient; unscheduled visits are set on demand in case of relapses or other medical emergencies related to the disease or its treatments. Moreover, a percentage of patients receive infusions of intravenous drugs regularly at the MS center. Before COVID-19 pandemic, any telemedicine programme was in place at our center, except for telephone consultation in case of urgent medical needs. In the period between March 2020 and May 2020, scheduled in-person visits were suspended for patients affected by chronic disorders, such as MS, due to the rapid surge of COVID-19 pandemic and in accordance with the recommendations of the Ministry of Health. Routine follow-up was managed by phone calls. In June 2020, in-person visits were allowed again, but there was still the need to reduce the number of neurological encounters in order to avoid patients’ gatherings. Clinicians were asked to identify those patients requiring or that would have benefitted from in-person visit and those that could be managed remotely. Against this setting, also considering the lack of objective methods (i.e. screening scale) to select patients eligible for telemedicine, the lack of information about the individual digital skills of our patients and the availability of broadband Internet at their home, the actual use of the Internet for non-medical reasons, and the familiarity with the most popular platforms for data sharing/social networking, we decided to create a survey to explore patients perception of telemedicine, and in particular televisit. We expected that these data would allow us to better tailor the offer of telemedicine to patients’ needs and individual profiles in order to efficiently incorporate telehealth services also in future clinical practice, outside the pandemic scenario.

### Survey

A 37-item questionnaire was created using the SurveyMonkey electronic platform (SurveyMonkey Inc., San Mateo, California, USA, www.surveymonkey.com). Full description of the survey is outlined in the supplementary materials (Online resource 1). A combination of forced choice (yes/no) and multiple-choice questions was used. The questionnaire was developed aiming to collect data regarding demographics, employment status and income, distance of the living place from the MS center, MS clinical characteristics, propensity to digital health, availability of digital devices at home and digital skills, previous experience with televisit and remote monitoring tools, as well as with the most common electronic platforms for data sharing (i.e. WhatsApp, Facebook). The link to the survey was sent by e-mail to 1098 patients with MS followed at the MS center of Tor Vergata Hospital, Rome, that have given their consent to the use of e-mail address according to the general European rules on data protection (GDPR). Questionnaire responses were anonymous, and there was no possibility to recognize the identity of respondents based on the answers. Respondents have been informed on the scope of the study and have decided freely to participate. As the first question respondents had to consent to the use of the information collected, otherwise the system would not allow the survey to continue. E-mails were sent out on July 24th, 2020, a reminder was sent 7 days later and the survey remained open until September 23rd, 2020.

### Statistical analysis

Categorical and continuous variables were presented respectively as N (%) and mean (SD). Logistic univariate regression models were used to evaluate the association of each variable with the propensity to telemedicine, and then, for each domain of the questionnaire, a multivariable logistic regression model was fitted including the variables resulting with a p value > 0.10 in the univariate analysis. Finally, a multivariable logistic regression model was fitted including all the variables which maintained a p value > 0.10 in the single-domain multivariable analysis.

Statistical analyses were performed using Stata version 15.1 (Stata Corporation, College Station, TX, USA).

## Results

**Fig. 1:**
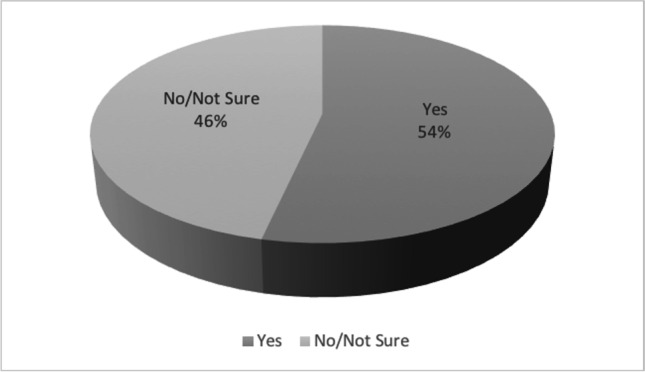
Propensity to televisit among those persons with MS completing the survey

A total of 613 out of 623 respondents completed the questionnaire giving consent to the use of their data. More than half of them (54%) declared to be open to telemedicine visit with neurologists of the MS center (Fig. 1).

Responders were mainly females (70%) and 55% of the individuals were 41–60 years old; 51% of the responders had a secondary school education level, 49% were employed, and 52% declared to have a medium income; among those employed, only 22% had a part-time job (Table 1).

**Table 1:** Demographic characteristics of responders and univariable/multivariable logistic regression models for the propensity to telemedicine. ** Logistic Regression model estimates refer to the change in the interest to telemedicine due to a unit class increase in the independent variable

Sociodemographic characteristics		Uni OR (95% CI)	*p* value	Multi OR (95% CI)	*p* value
Sex, N (%)					
Male	170 (30%)	1.15 (0.80–1.66)	0.439		
Female	400 (70%)	1.00 (ref)	-—-		
Age, N (%)					
18–30 years	50 (9%)				
31–40 years	138 (24%)	-—-	-—-		
41–50 years	185 (32%)	-—-	-—-		
51–60 years	132 (23%)	-—-	-—-		
61–70 years	57 (10%)	-—-	-—-		
> 70 years	8 (1%)	0.94 (0.81–1.08)	0.365**		
Education, N (%)					
Elementary school	7 (1%)	-—-	-—-		
Middle school	80 (14%)	-—-	-—-		
Secondary school	289 (51%)	-—-	-—-		
Degree	153 (27%)	-—-	-—-		
Postgraduate education	41 (7%)	1.83 (1.46–2.27)**	< 0.001**	1.59 (1.25–2.02)**	< 0.001**
Occupational status**,** N (%)					
Unemployed	110 (20%)	1.00 (ref)	-—-		
Retired	98 (18%)	1.10 (0.63–1.90)	0.741	0.85(0.46–1.57)	0.598
Student	19(3%)	1.78 (0.66–4.76)	0.253	1.20 (0.43–3.36)	0.725
Self-employed	61(11%)	1.99 (1.05–3.77)	0.034	0.99 (0.48–2.04)	0.974
Employed	272(49%)	2.45 (1.56–3.84)	< 0.001	1.34 (0.76–2.38)	0.315
Contract type, N (%)					
Part-time	68 (22%)	1.00 (ref)			
Full-time	243 (78%)	1.01 (0.58–1.76)	0.983		
Income, N (%)					
No income	84 (15%)	-—-	-—-		
Low	174 (31%)	-—-	-—-		
Medium	294 (52%)	-—-	-—-		
High	18 (3%)	1.65 (1.32–2.06) **	< 0.001**	1.38 (1.04–1.83)**	0.027**

Demographic characteristics that significantly increased the interest in telemedicine were having a higher education level (*p* < 0.001) or income (*p* < 0.001) and being self-employed (*p* = 0.034) or employed (*p* < 0.001) compared to being unemployed. However, when the demographics multivariable analysis was run, only education and income remained significant (education, *p* < 0.001; income, *p* = 0.027).

Concerning MS characteristics, most of the participants were taking disease-modifying treatments (84%), did not need any walking assistance (75%), and had been diagnosed with MS more than five years before (80%) (Table 2). We did not find any association between MS characteristics and propensity to telemedicine (treatment, *p* = 0.147; years after diagnosis, *p* = 0.859), also including the degree of motor disability (*p* = 0.181; *p* = 0.667; *p* = 0.578).

**Table 2:** MS characteristics of responders and univariable/multivariable logistic regression models for the propensity to telemedicine. ** Logistic Regression model estimates refer to the change in the interest to telemedicine due to a unit class increase in the independent variable

MS characteristics		OR (95% CI)	*p* value
Mobility, N (%)			
Without help	397 (75%)	1.00 (ref)	-—-
Unilateral assistance	64 (12%)	0.70 (0.41–1.18)	0.181
Bilateral assistance	31 (6%)	0.85 (0.40–1.78)	0.667
Wheelchair use	36 (7%)	1.23 (0.60–2.53)	0.578
Therapy, N (%)			
No	86 (16%)	1.43 (0.88– 2.34)	0.147
Yes	457 (84%)	1.00 (ref)	-—-
Disease duration, N (%)			
< 1 year	19 (3%)	-—-	-—-
1–4 years	95 (17%)	-—-	-—-
5–9 years	140 (26%)	-—-	-—-
10–19 years	220 (41%)	-—-	-—-
≥ 20 years	69 (13%)	1.02 (0.86–1.20) **	0.859**

Regarding the access to the MS center, most of the respondents lived within a 50-km radius from the MS center (74%), and only 23% of them needed a caregiver to reach the MS center due to neurological deficits (Table 3). According to the multivariable analysis, respondents more interested in telemedicine were those who lived farther from the center compared to individuals living closer (*p* = 0.032) and those usually reaching the MS center alone compared to individuals needing aid (*p* = 0.007).

**Table 3:** Characteristics of the Responders’ access to the MS center and univariable/multivariable logistic regression models for the propensity to telemedicine. ** Logistic Regression model estimates refer to the change in the interest to telemedicine due to a unit class increase in the independent variable

Logistic characteristics					
		OR (95% CI)	*p* value	OR (95% CI)	*p* value
Distance from the MS center, N (%)					
< 10 km	99 (18%)	-—-	-—-		
10–50 km	302 (56%)	-—-	-—-		
50–100 km	86 (16%)	-—-	-—-		
> 100 km	56 (10%)	1.21 (0.98–1.48)**	0.076**	1.26 (1.02–1.56)**	0.032**
Time required, N (%)					
< 1 h	62 (12%)	-—-	-—-		
1–2 h	172 (32%)	-—-	-—-		
3–4 h	224 (42%)	-—-	-—-		
> 4 h	80 (15%)	1.02 (0.83–1.24)**	0.865**		
Time off work, N (%)					
No	146 (39%)	1.00 (ref)	-—-		
Yes, ½ day	75 (20%)	1.01 (0.56–1.82)	0.969		
Yes, 1 day	144 (39%)	1.22 (0.75–1.99)	0.428		
Yes, > 1 day	6(2%)	2.68 (0.31–23.60)	0.373		
Accompanying person, N (%)					
No	111 (21%)	1.99 (1.16–3.40)	0.012	2.13 (1.23–3.66)	0.007
Yes, but not necessary/sometimes	305 (57%)	1.37 (0.90–2.09)	0.145	1.38 (0.90–2.11)	0.139
Yes, help necessary	122 (23%)	1.00 (ref)	-—-	1.00(ref)	-—-
Mean of transportation used to reach the center, N (%)					
Private	467 (87%)	1.00 (ref)	-—-		
Public	69 (13%)	1.31 (0.77–2.24)	0.319		

Almost all the participants had Internet at home (92%), used Internet (94%), and e-mails (90%) regularly (Table 4). However, only 59% of them had used Internet for remote activities, and the percentage of patients who used technologies to monitor workout performance was < 15%. Concerning the sharing online platforms they were familiar with, most of the respondents reported to use WhatsApp (82%), while only 21% and 12% Zoom and Teams. According to the univariate logistic regression model, propensity to telemedicine depended on access to Internet at home (*p* = 0.008), habitual use of Internet (*p* = 0.001), routine use of computer or tablet (*p* < 0.001), use of Internet for other remote activities (*p* < 0.001), use of e-mails (*p* < 0.001), and use of electronic technologies to monitor workout performances (*p* = 0.016). However, in the multivariable model, only the use of a computer or tablet (*p* = 0.003) and the use of Internet for remote activities (*p* < 0.001) were found statistically significant.

**Table 4:** Responders’ attitude towards the use of Internet and technologies and univariable/multivariable logistic regression models for the propensity to telemedicine

Internet and technologies characteristics		OR (95% CI)	*p* value	OR (95% CI)	*p* value
Internet available at home, N (%)					
Yes	491(92%)	2.32 (1.25–4.32)	0.008	1.28(0.55–2.96)	0.568
No	45(8%)	1.00(ref)	-—-		
Habitual use of Internet, N (%)					
Yes	501 (94%)	3.82 (1.77–8.25)	0.001	0.68 (0.24–1.88)	0.453
No	32 (6%)	1.00 (ref)	-—-		
Technologies used, N (%)					
None or only smartphone	220 (36%)	1.00 (ref)	-—-		
Also tablet or/and computers	393 (64%)	6.82 (4.70–9.91)	< 0.001	1.90 (1.06–3.38)	0.003
Use of Internet for remote activities, N (%)					
No	213 (41%)	1.00 (ref)	-—-		
Yes	312 (59%)	4.40 (3.02–6.41)	< 0.001	3.09 (1.98–4.85)	< 0.001
Use of Internet for medical information, N (%)					
No	66 (13%)	1.00 (ref)	-—-		
Yes	459 (87%)	1.68 (1.00–2.82)	0.051	1.37 (0.72–2.58)	0.335
Use of e-mail, N (%)					
No	54 (10%)	1.00 (ref)	-—-		
Yes	471 (90%)	4.23 (2.31–7.75)	< 0.001	1.80 (0.80–4.04)	0.154
Use of technologies to monitor workout performance					
No	406 (86%)	1.00 (ref)	-—-		
Yes	68 (14%)	2.07 (1.14–3.75)	0.016	1.27 (0.67–2.40)	0.468

When all the variables showing a *p* value < 0.10 in the single-domain multivariable logistic models were included in the all-domains multivariable model, the propensity to telemedicine significantly depended on having a higher income (*p* = 0.037), living farther from the center (*p* = 0.038), using computer and tablet (*p* = 0.010), and using Internet for other remote activities (*p* < 0.001).

More than half of the respondents did not know the meaning of the word ‘telemedicine’ before the COVID-19 pandemic (Table 5). The main advantages and disadvantages of telemedicine reported by respondents were saving time (70%) and limited possibility to properly assess the neurological status (71%), respectively. When respondents were asked to evaluate the anticipated perceived accuracy of the televisit on a 0–4 rating scale, the mean score was 2.1(± 1.0, range 0–4).

**Table 5:** Responder’s knowledge and opinions about telemedicine

Telemedicine knowledge and general opinion	
Knowledge of the telemedicine before the COVID-19 pandemic, N (%)	
Yes	225 (43%)
No	300 (57%)
Main advantage of telemedicine, N (%)	
Saving time	327 (70%)
Saving money	47 (10%)
No accompanying persons needed	91 (20%)
Main disadvantage of telemedicine, N (%)	
Difficulties in using technologies due to MS	27 (6%)
Difficulties in using technologies in general	40 (8%)
Lack of technological support	6 (1%)
Difficulties in fully explaining health problems	64 (14%)
No possibility of assessing neurological status	337 (71%)
0–4 score evaluation of utility and completeness of telemedicine, mean (SD)	2.1 (1.0)
Preference of telemedicine compared to telephone contact, N (%)	
Yes	278 (82%)
No	62 (18%)
Preference of telemedicine compared to visits at the center, N (%)	
Yes, always	10 (2%)
Yes, but periodically	78 (17%)
Yes, but occasionally	231 (51%)
No	138 (30%)
When telemedicine can replace visits at the center	
Normal FU for exams monitoring, N (%)	251 (81%)
Assistance for the onset of a new symptom or for urgences, N (%)	132 (43%)
Evaluation of therapy change, N (%)	95 (31%)
Multidisciplinary visit, N (%)	52 (17%)
For patients from a different region, N (%)	113 (37%)
For patients with mobility problems, N (%)	131 (42%)
Open to use technologies to evaluate neurological measurements during a telemedicine visit, N (%)	
No	132 (28%)
It depends on the cost and on difficulty in their use	120 (25%)
Yes	222 (47%)
Open to use a Web App to update personal information, information about therapies, exams and visits reports, N (%)	
No	54 (14%)
Yes	321 (86%)
Evaluation of telemedicine, if experienced, N (%)	
Not able to connect	33 (52%)
Assistance needed	4 (6%)
Some problems but solved in autonomy	6 (9%)
No problems	21 (33%)
0–10 score evaluation of telemedicine, if experienced, mean (SD)	6.7 (2.9)

Most of the individuals (82%) declared that they would prefer telemedicine to telephone contact. For only 2% of the respondents, telemedicine visit should replace all in-person visits, while for 81% it is suitable only for routine clinical follow-up, for around 40% for assistance in case of MS relapse or other emergencies, and for patients with limited mobility and for patients living in other Italian regions. Only 17% of responders reported that telemedicine can be useful when multidisciplinary consultation is required.

Referring to the use of electronic devices for remote neurological monitoring, 47% of the individuals were definitely open to using them, while 25% were unsure due to the cost and limited confidence with these tools. Interestingly, most of the participants (86%) would agree to update their personal and medical information using a Web App.

Only 64 respondents had already experienced a telemedicine visit before the date of the questionnaire, and even if the mean score for the visit evaluation was slightly over the sufficiency (mean = 6.7, SD = 2.9), more than half of the individuals failed to connect with the neurologist due to technological glitches, and only 27 (42%) of them did not need assistance during the telemedicine visit.

## Discussion

The COVID-19 pandemic is speeding up the digital transformation of the healthcare system that has already started in the pre-pandemic epoch. Remote patient monitoring and televisit represent only the ‘tip of the iceberg’ of such global reorganization and in particular the reorganization of neurology into teleneurology [11].

In order to develop digital solutions that are efficient, useful, and sustainable, it is indispensable to listen to patients’ opinions and explore their attitudes.

In this study we have asked to a large sample of patients with MS their opinion on the use of televisit for the monitoring of their disease; moreover, we have explored the sociodemographic and clinical characteristics, as well as digital and technological skills of respondents, orienting their propensity to televisit.

Although the slight majority of MS patients were in favour of televisit, there is still a large group of people that refuse it. Nevertheless, the majority of the respondents declared that they did not know the meaning of ‘telemedicine’ before the pandemic, suggesting that educating patients on the new digital healthcare tools is of utmost importance.

In support of this observation, our results show that highly educated persons were significantly more interested in telemedicine. Surprisingly the age of respondents was irrelevant and old people were as open as young to telemedicine. Unfortunately, due to the higher prevalence of MS among young middle-aged people, the sample size of respondents older than 70 years old was very small; therefore the interpretation of the results in this population is limited.

Counterintuitively, none of the MS clinical characteristics was associated with higher or lower propensity to televisit and in particular the level of disability. Neither the use of bilateral assistance or wheelchair nor the need of a caregiver accompanying the person with MS to the visit increased the propensity to televisit. It is commonly believed that one of the most important advantages of telemedicine in the field of neurology and MS is delivering care to fragile people with mobility impairment [12, 13]. However, our data does not completely confirm this a priori assumption; we speculated that patients with higher disability, notwithstanding the potential risk of contagious or higher costs, would rather prefer face-to-face encounters as they allow a more complete neurological exam (i.e. in case of spasticity or multifunctional impairment). The limited possibility of assessing neurological status during remote visit was, in fact, identified by the majority of the respondents as one the most important disadvantages of such approach. Several tools and methods are now available to overcome this barrier and improve teleneurology practice [14–16], but patients and neurologists need time to get familiar with them and increase the level of trust in these approaches [17].

Another anticipated advantage of telemedicine is the possibility to reach persons living in rural areas underserved by medical assistance [18]. In Italy many persons with MS are currently followed up in MS centers that are very far from their living place, due to the lack of qualified local neurological assistance. Expectedly, according to our results, patients living farther (> 100 km) from the MS center were more open to remote visits compared to those living nearby.

Such findings have important implications in the role of the MS center; they open the way to increase the reach also of such patients living in remote Italian regions, that, due to poor personal, financial or social resources, are currently underdiagnosed or under treated, ensuring equitable care access for all. Moreover, these data support the possibility to monitor more accurately geographically distant patients, also in the case of suspected relapse [16].

Indeed, the majority of the respondents, independently from where they live, identified routine monitoring visits and assistance for suspected disease reactivation as the most suitable indications for occasional televisit.

The positive aspect emerging from our survey is that almost all the respondents have Internet connection at home and use e-mails. Nevertheless, as these data were collected through an electronic web- based survey, it is also possible that we have pre-selected a sample of patients with more digital skills, limiting the validity of our results. However, about 40% of the respondents do not have a pc/tablet and use smartphones for online activities. This should be taken into account in order to increase the usability of telemedicine platforms by all the users [19].

Indeed, among those few people that had already tried the experience of televisit, less than 50% were able to connect with the neurologist, and their level of satisfaction was barely sufficient, indicating that either the platforms used were not easily accessible or that patients need to be better trained to use them. Initiatives to improve digital patients’ literacy and special support programmes should be put in place. Several pieces of evidence have highlighted that differences in the level of digital expertise may increase the digital divide among patients [10, 20]; a recent study in patients with dementia has shown that prevalence of successful televisit was higher in the presence of younger caregivers with higher experience with technology [10].

Interestingly, our results showed that higher income, independently from the type of employment contract, was also associated with higher propensity to televisit. Consistently, one of the main foreseen advantages of telemedicine was saving time (i.e. for travel), more than saving money, as already reported [21].

In conclusion, telemedicine is a viable approach for patients with MS during and outside pandemic due to its potential in complementing in person assistance in the continuous monitoring of patients with chronic medical conditions, like MS, especially if they live far from the hospital.

However, there are still several general and specific barriers limiting the wide use of these approaches in the MS field at the moment: the digital divide among patients, lack of adequate digital tools tailored for persons with MS patients, and poor trust in the online evaluation of the neurological status.

We acknowledge that results of this survey may be partially influenced by the pandemic emergency, although responses have been collected in a period in which in-person visits were allowed again. In order to confirm patients’ propensity toward telemedicine, it is advisable to explore and monitor their opinion and adherence to telemedicine longitudinally and in particular outside pandemic. Telemedicine services would benefit from initiatives such as feedback systems where patients can give ratings to their televisit experience and elaborate suggestions on how to improve the service.

## Supplementary Information

Below is the link to the electronic supplementary material.

Supplementary file1 (DOCX 11 KB)

## Data Availability

The datasets generated during and/or analysed during the current study are available from the corresponding author on reasonable request.

## References

[1] Danhieux K, Buffel V, Pairon A (2020). The impact of COVID-19 on chronic care according to providers: a qualitative study among primary care practices in Belgium. BMC Fam Pract.

[2] Courtney E, Blackburn D, Reuber M (2021) Neurologists’ perceptions of utilising tele-neurology to practice remotely during the COVID-19 pandemic. Patient Education and Counseling S0738399120306911. 10.1016/j.pec.2020.12.027 10.1016/j.pec.2020.12.027 33478853

[3] Gentry MT, Puspitasari AJ, McKean AJ, et al (2021) Clinician satisfaction with rapid adoption and implementation of telehealth services during the COVID-19 pandemic. Telemedicine and e-Health tmj.2020.0575. 10.1089/tmj.2020.0575 10.1089/tmj.2020.0575 33606560

[4] Kristoffersen ES, Sandset EC, Winsvold BS (2021). Experiences of telemedicine in neurological out-patient clinics during the COVID-19 pandemic. Ann Clin Transl Neurol.

[5] Lahat A, Shatz Z (2021). Telemedicine in clinical gastroenterology practice: what do patients prefer?. Therap Adv Gastroenterol.

[6] Rametta SC, Fridinger SE, Gonzalez AK (2020). Analyzing 2,589 child neurology telehealth encounters necessitated by the COVID-19 pandemic. Neurology.

[7] Riley PE, Fischer JL, Nagy RE (2021). Patient and provider satisfaction with telemedicine in otolaryngology. OTO Open.

[8] Sathiyaraj A, Lopez H, Surapaneni R (2021) Patient satisfaction with telemedicine for prechemotherapy evaluation during the COVID-19 pandemic. Future Oncology fon-2020–0855. 10.2217/fon-2020-0855 10.2217/fon-2020-0855 PMC7909235 33631995

[9] von Wrede R, Moskau-Hartmann S, Baumgartner T (2020). Counseling of people with epilepsy via telemedicine: experiences at a German tertiary epilepsy center during the COVID-19 pandemic. Epilepsy Behav.

[10] Arighi A, Fumagalli GG, Carandini T (2021). Facing the digital divide into a dementia clinic during COVID-19 pandemic: caregiver age matters. Neurol Sci.

[11] Hatcher-Martin JM, Adams JL, Anderson ER (2020). Telemedicine in neurology: telemedicine work group of the american academy of neurology update. Neurology.

[12] Yeroushalmi S, Maloni H, Costello K, Wallin MT (2020). Telemedicine and multiple sclerosis: a comprehensive literature review. J Telemed Telecare.

[13] Kang PB, Bale JF, Mintz M (2016). The child neurology clinical workforce in 2015: report of the AAP/CNS Joint Taskforce. Neurology.

[14] Romeo AR, Rowles WM, Schleimer ES, et al (2020) An electronic, unsupervised patient-reported expanded disability status scale for multiple sclerosis. Mult Scler 135245852096881. 10.1177/1352458520968814 10.1177/1352458520968814 PMC8144241 33236967

[15] Bove R, Bevan C, Crabtree E (2019). Toward a low-cost, in-home, telemedicine-enabled assessment of disability in multiple sclerosis. Mult Scler.

[16] on behalf of the Digital Technologies Web and Social Media Study Group of the Italian Society of Neurology, Moccia M, Lanzillo R, et al (2020) Assessing disability and relapses in multiple sclerosis on tele-neurology. Neurol Sci 41:1369–1371. 10.1007/s10072-020-04470-x 10.1007/s10072-020-04470-x PMC7241064 32440979

[17] Allen-Philbey K, Middleton R, Tuite-Dalton K (2020). Can we improve the monitoring of people with multiple sclerosis using simple tools, data sharing, and patient engagement?. Front Neurol.

[18] Dorsey ER, Topol EJ (2016). State of telehealth. N Engl J Med.

[19] Agha Z, Weir CR, Chen Y (2013). Usability of telehealth technologies. Int J Telemed Appl.

[20] Eberly LA, Khatana SAM, Nathan AS (2020). Telemedicine outpatient cardiovascular care during the COVID-19 pandemic: bridging or opening the digital divide?. Circulation.

[21] Nanda M, Sharma R (2021) A review of patient satisfaction and experience with telemedicine: a virtual solution during and beyond COVID-19 pandemic. Telemedicine and e-Health tmj.2020.0570. 10.1089/tmj.2020.0570 10.1089/tmj.2020.0570 33719577

