# Supplementary material for: Patient’s point of view on the use of telemedicine in multiple sclerosis: a web-based survey
Source: Neurol Sci. 2021 Jul 20;43(2):1197–205. doi: 10.1007/s10072-021-05398-6 (PMC8289711; doi:10.1007/s10072-021-05398-6)
Supplement: Supplementary file 1 — Supplementary file1 (DOCX 11 KB) [file 10072_2021_5398_MOESM1_ESM.docx]

**Survey**

1. Do you agree to participate in the study? Yes/No.
2. Are you a male or a female? Male/Female.
3. What is your age? 18-30/31-40/41-50/51-60/61-70/>70 years.
4. What is the highest degree or level of school you have completed? Elementary School/ Middle School/Secondary School/ Degree/ Postgraduate education.
5. Are you currently…? Unemployed/ Retired/ Student/Self-employed/Employed/ Other.
6. If you are employed, do you work part-time or full-time? Part Time/Full Time/Not working/Other.
7. Rate your total household income. No income/Low/Medium/High
8. Do you need assistance for walking? No/ Yes, unilateral assistance/ Yes, bilateral assistance/ Yes, I use a wheelchair/ Other.
9. Are you under treatment for MS? If yes, please provide the name of the treatment.
10. How many years ago were you diagnosed with MS? <1/ 1-4/ 5-9/ 10-19/ >=20 years ago.
11. How far is your home from the MS center of Tor Vergata? <10 Km/ 10-50 Km/50-100 Km/ >100 Km.
12. On average, how much time do you spend for a neurological checkup visit at the MS center? <1h /1-2h/ 3-4h/ >4h.
13. Do you need to ask the employer for permission for a checkup visit? No/ Yes, ½ day/ Yes, 1 day/ Yes, >1 day/ Not working.
14. Do you usually need help from someone else (accompanying person) to reach the MS center? No/ Yes, sometimes/ Yes, but not necessary/ Yes, help necessary.
15. Which means of transport do you use to reach the MS center? Plane/ Train/ Car/ Bus/ On foot/ Other.
16. Do you have an internet connection at home? Yes/No
17. Do you habitually use the Internet? Yes/No
18. If you use the Internet, what digital devices do you use? Smartphone/ Tablet/ Personal Computer/ Work Computer/ Other.
19. Have you ever used the Internet for remote activities? Yes, for lessons/ Yes, for Sport/ Yes, for working activities/ No.
20. Have you ever used the Internet for health reasons? Yes, to learn more about MS/ Yes, to learn more about other health aspects/ Yes, to find a specialist/ Yes, to schedule an appointment/ No
21. Do you habitually use the e-mail? Yes/No
22. Which e-mail address do you use? Personal/ Work / Relative’s address/ Friend’s Address.
23. Which social networks or video communication platforms are you familiar with? Skype/ WhatsApp/ Zoom/ Google Meet/ Teams/ FaceTime/ None.
24. Before today, did you know the meaning of the word “Telemedicine”? Yes/ No.
25. Are you open to have a telemedicine visit with the neurologist of the MS center? Yes, I have already experienced a Telemedicine Visit/ Yes, I would like to/ No, not interested/ Not sure.
26. If you have already experienced a telemedicine visit, how was your experience? No connection problems/ Some connection problems but solved in autonomy/ Assistance Needed/ Not able to connect.
27. If you have already experienced a telemedicine visit, rate your level of satisfaction: 1-10
28. In your opinion, which is the main advantage of Telemedicine? Saving Time/ Saving Money/ No accompanying persons needed/ Other.
29. In your opinion, which is the main disadvantage of Telemedicine? Difficulties in technologies use due to MS/ Difficulties in technologies use in general/ Lack of technological support/ Difficulties in clearly explaining health problems/ No possibility of assessing neurological status/ Other.
30. Do you think that a Telemedicine visit is complete? Disagree/ Poorly agree/ Sufficiently agree/ Agree/ Greatly agree.
31. In your opinion, a telemedicine visit is preferable to a phone contact? Yes/ No/ Not sure.
32. If you think that a phone contact is better than a telemedicine visit, please provide the motivations supporting your opinion.
33. In your opinion, can a telemedicine visit replace a visit at the MS center? Yes, always/ Yes, but periodically/ Yes, but occasionally/ No/ Not sure.
34. In your opinion, in what situations can the visit at the center be replaced by the telemedicine? Normal FU for exams monitoring/ Assistance for the onset of a new symptom or for emergencies/ Evaluation of therapy change/ Multidisciplinary consulting visit/ For patients from a different region/ For patients with mobility problems/ Other.
35. Do you use technologies to measure your mobility performance? Yes/ No
36. Would you use digital technologies to evaluate neurological status during a telemedicine visit? Yes/ No/ It depends on the cost and on difficulty in their use.
37. Are you open to use a Web App to update personal information, information about therapies, exams, and visits reports? Yes/ No/ Not sure.
